# Cullin 4A (CUL4A), a direct target of miR-9 and miR-137, promotes gastric cancer proliferation and invasion by regulating the Hippo signaling pathway

**DOI:** 10.18632/oncotarget.7048

**Published:** 2016-01-28

**Authors:** Jun Deng, Wan Lei, Xiaojun Xiang, Ling Zhang, Jun Lei, Yu Gong, Meijiao Song, Yi Wang, Ziling Fang, Feng Yu, Miao Feng, Ze Sun, Jun Chen, Zhengyu Zhan, Jianping Xiong

**Affiliations:** ^1^ Department of Oncology, The First Affiliated Hospital of Nanchang University, Nanchang 330006, PR China

**Keywords:** CUL4A, Hippo, miR-9, miR-137, gastric cancer

## Abstract

Although Cullin 4A (CUL4A) is mutated or amplified in several human cancer types, its role in gastric cancer (GC) and the mechanisms underlying its regulation remain largely uncharacterized. In the present study, we report that the expression of CUL4A significantly correlated with the clinical stage of the tumor and lymph node metastasis, and survival rates were lower in GC patients with higher levels of CUL4A than in patients with lower CUL4A levels. The upregulation of CUL4A promoted GC cell proliferation and epithelial-mesenchymal transition (EMT) by downregulating LATS1-Hippo-YAP signaling. Knocking down CUL4A had the opposite effect *in vitro* and *in vivo*. Interestingly, CUL4A expression was inhibited by the microRNAs (miRNAs), miR-9 and miR-137, which directly targeted the 3′-UTR of CUL4A. Overexpression of miR-9 and miR-137 downregulated the CUL4A-LATS1-Hippo signaling pathway and suppressed GC cell proliferation and invasion *in vitro*. Taken together, our findings demonstrate that perturbations to miR-9/137-CUL4A-Hippo signaling contribute to gastric tumorigenesis, and suggest potential therapeutic targets for the future treatment of GC.

## INTRODUCTION

Gastric cancer (GC) is the fourth most common malignant carcinoma and ranks second worldwide as a cause of cancer-related deaths [1]. The incidence of GC is the highest in China, accounting for nearly 42% of cases[2]. Despite advances in surgical, chemotherapeutic, radiation, and anti-ERBB2 molecular targeted therapies, 5-year survival rates have improved only minimally during the past few decades. Although mutations and alterations in a large number of oncogenes and cancer suppressor genes have been identified in gastric carcinomas, the molecular mechanisms underlying GCs are still poorly understood.

Cullin 4A (CUL4A) is an E3 ubiquitin ligase that regulates several well-defined tumor suppressor genes, such as p53, p73, and CDKN1B (p27)[3–5]. Besides being involved in the ubiquitin-proteasome pathway, CUL4A is a transcriptional co-activator mediating EGFR and ZEB1 activation, which can promote cancer cell proliferation and EMT through epigenetic mechanisms [6, 7]. Recent data support that the high expression of CUL4A is associated with adverse clinical outcomes in several cancer types, including breast, lung, and bone cancer [6–8]. However, the involvement of CUL4A in GC and the mechanisms that regulate its expression remain poorly characterized.

MicroRNAs (miRNAs) constitute a class of small non-coding RNAs (19-24 nucleotides) that regulate gene expression post-transcriptionally by targeting the 3′ untranslated regions (3′-UTRs) of expressed mRNAs [9–11]. In GC, oncogenic miRNAs such as miR-21 [12], miR-362 [13] and miR-296-5p [14] are abnormally upregulated, and tumor suppressing miRNAs such as miR-506 [15], miR-129-5p [16] and miR-361-5p [17] are significantly downregulated. However, whether miRNAs can regulate the expression of CUL4A has not been reported.

In the present study, we show that CUL4A expression is correlated with tumor-node-metastasis (TNM) clinical stages of GC. In addition, we demonstrate that CUL4A promotes GC proliferation, EMT, and invasion by inactivating the Hippo signaling pathway. We also identified that miR-137 and miR-9 directly downregulate CUL4A expression by targeting the 3′-UTR of its mRNA, and indirectly regulate downstream Hippo-YAP signaling in GC. Taken together, our findings demonstrate the importance of a miR-9/137-CUL4A-Hippo signaling axis in GC, and suggest new therapeutic targets for future treatment of GC.

## RESULTS

### CUL4A expression is upregulated in GC tissues and cell lines

**Figure 1:**
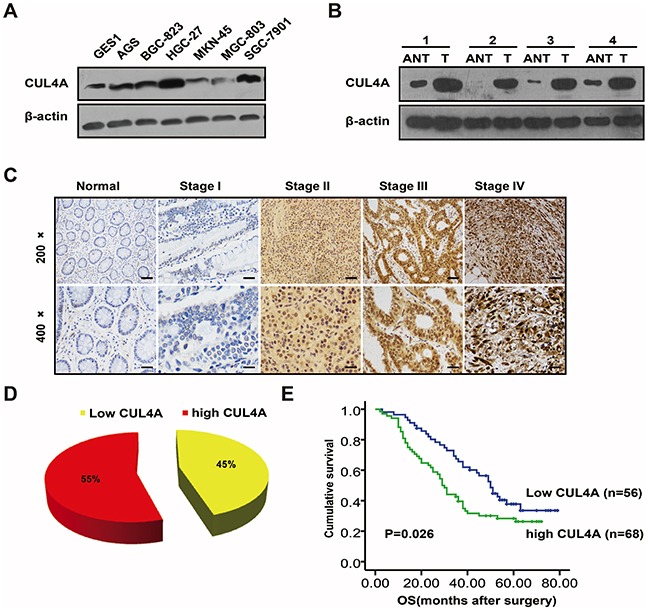
Expression of CUL4A is upregulated in gastric cancer tissues and cell lines **A.** Western blotting results of CUL4A protein levels in different GC cell lines. CUL4A protein levels are higher in the six GC cell lines than in the human immortalized gastric epithelial cell line (GES-1). **B.** Western blotting results of CUL4A protein levels in GC tissues and adjacent noncancerous gastric tissues. CUL4A protein levels are higher in GC tissues (T) than in adjacent noncancerous gastric tissues (ANT) from the same patient (*n*=4). **C.** IHC staining of CUL4A protein in human GC (clinical stages I-IV) and normal gastric tissues. **D.** GC patient samples were stratified by CUL4A protein expression into low (45%) and high (55%) groups (*n*=124). **E.** Kaplan-Meier survival curves indicated that higher levels of CUL4A (*n*=68) are associated with lower patient survival rates than lower levels of CUL4A (*n*=56). Each bar represents the mean±SD of three independent experiments. ^*^p-value <0.05.

Overexpression of CUL4A has been reported in several types of human cancers [18]; however, its expression level in GC has not been characterized. The protein levels of CUL4A in samples prepared from several GC cell lines and the human immortalized gastric epithelial cell line (GES-1) were analyzed by western blotting. Compared to the GES-1 cell line, all cancer cell lines expressed higher levels of CUL4A protein (Figure 1A). Moreover, as determined by western blotting, CUL4A protein levels were higher in gastric tumors than in adjacent noncancerous gastric tissues (*n*=4) (Figure 1B).

Higher levels of CUL4A protein in gastric tumors compared to normal gastric mucosa were also observed by immunohistochemistry. CUL4A protein was either not detected (16/20; 80%) or only marginally detected (4/20; 20%) in noncancerous gastric tissues (data not shown). In contrast, in gastric tumors, higher levels of CUL4A protein were detected (68/124; 55%) (Figure 1D), and CUL4A was distributed nucleocytoplasmically. Results from western blotting and immunohistochemistry demonstrate that higher CUL4A protein expression is associated with GC.

Next, we correlated CUL4A protein expression with the clinical stages of GC. As shown in Supplementary Table S1, CUL4A protein levels strongly correlated with TNM stage (P=0.025) and lymph node metastasis (P=0.003) (Figure 1C). However, no significant difference in CUL4A expression was observed due to gender, age, differentiation or tumor size. In addition, compared to GC patients with lower CUL4A levels, GC patients with higher CUL4A levels had lower survival rates (Figure 1E). These results suggest that CUL4A may function as an oncogene in GC cells.

### CUL4A promotes GC cell proliferation

**Figure 2:**
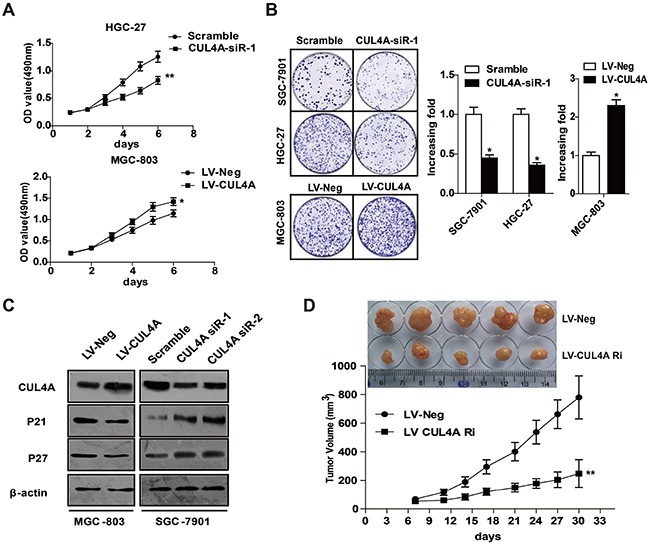
CUL4A promotes GC cell proliferation **A.** MTT assay results after knocking down or overexpressing CUL4A. Knocking down CUL4A inhibited cell proliferation of HGC-27 cells compared to cells in the control mock-treated group; whereas, overexpressing CUL4A enhanced MGC-803 cell proliferation. **B.** Clone formation assay results after knocking down or overexpressing CUL4A. Knocking down CUL4A suppressed SGC-7901 and HGC-27 cell proliferation in clone formation assays; whereas, MGC-803 cell proliferation was enhanced by overexpressing CUL4A. **C.** Knocking down CUL4A resulted in the upregulation of p21 and p27 in SGC-7901 cells; whereas, overexpressing CUL4A led to opposite results in MGC-803 cells. **D.** Representative images of tumors from mice in each group. Tumor volumes were measured on the indicated days. Each bar represents the mean±SD of three independent experiments. ^*^p-value <0.05.

To investigate the role of CUL4A in GC cell proliferation, we conducted loss-of-function and gain-of-function experiments. Importantly, the up- or down-regulation of CUL4A protein was verified in all experiments in which GC cell phenotypes were characterized (Figure 2C and Figure 4B). MTT assays showed that HGC-27 cell proliferation was significantly suppressed after CUL4A downregulation, whereas MGC-803 cell proliferation was enhanced after CUL4A overexpression (Figure 2A). Furthermore, clone formation assays also showed that overexpressing CUL4A promoted GC cell proliferation (Figure 2B), which is consistent with our MTT assay results. In addition, since p21 and p27 play vital roles in cell proliferation and cell cycle progression, we measured p21 and p27 protein levels after CUL4A down- or up-regulation. Consistent with results in a previous study, we found that knocking down CUL4A resulted in the upregulation of p21 and p27 in SGC-7901 cells; whereas, overexpressing CUL4A led to opposite results in MGC-803 cells (Figure 2C). Furthermore, we designed a lentivirus vector to decrease CUL4A protein expression in HGC-27 cells in order to determine the role of CUL4A in the growth of GC xenografts in nude mice. Compared to a negative control lentivirus, lentivirus-mediated knockdown of CUL4A significantly inhibited growth of HGC-27 tumor xenografts (Figure 2D). Taken together, these results show that CUL4A promotes GC cell proliferation.

### CUL4A promotes GC invasion and EMT

**Figure 3:**
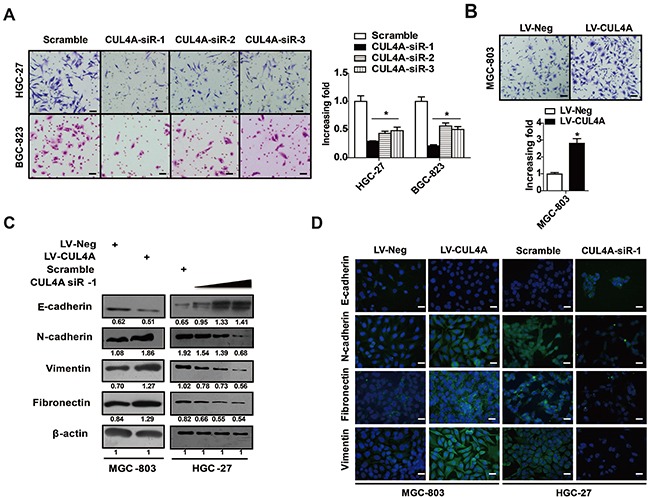
CUL4A promotes GC invasion and EMT **A.** Knockdown of CUL4A with a set of 3 siRNAs significantly inhibited HGC-27 and BGC-823 cell invasion. **B.** Overexpressing CUL4A promoted MGC-803 cell invasion. **C.** Knockdown of CUL4A with siR-1 (10 nM, 20 nM, 50 nM) increased E-cadherin expression, and inhibited N-cadherin, fibronectin and vimentin protein expression in HGC-27 cells. Conversely, overexpressing CUL4A led to opposite results in MGC-803 cells. **D.** Immunofluorescence results for EMT markers after GC cells were transfected with CUL4A-siR-1 or infected with lentivirus overexpressing CUL4A. Each bar represents the mean±SD of three independent experiments. ^*^p-value <0.05.

Invasion and metastasis are major hallmarks of cancer progression. We showed that CUL4A protein expression was significantly correlated with lymph node metastasis in GC patients. However, only a few studies have examined whether CUL4A regulates cancer cell invasion. In the present study, we demonstrate that downregulation of CUL4A by a set of 3 siRNAs significantly inhibited HGC-27 and BGC-823 cell invasion (Figure 3A), whereas overexpressing CUL4A had the opposite effect on MGC-803 cell invasion (Figure 3B). Epithelial-mesenchymal transition (EMT) is a major process in the initiation of cancer cell invasion; therefore, we tested whether the up- or down-regulation of CUL4A resulted in changes to EMT markers. As assayed by western blotting, the downregulation of CUL4A resulted in an increase in E-cadherin protein expression and decreases in N-cadherin, fibronectin and vimentin protein expression in HGC-27 cells (Figure 3C). The overexpression of CUL4A promoted EMT in MGC-803 cells (Figure 3C). These results were further confirmed by immunofluorescence (Figure 3D). Collectively, these results suggest that CUL4A promotes GC invasion and EMT, in addition to proliferation.

### CUL4A regulates Hippo-YAP signaling

**Figure 4:**
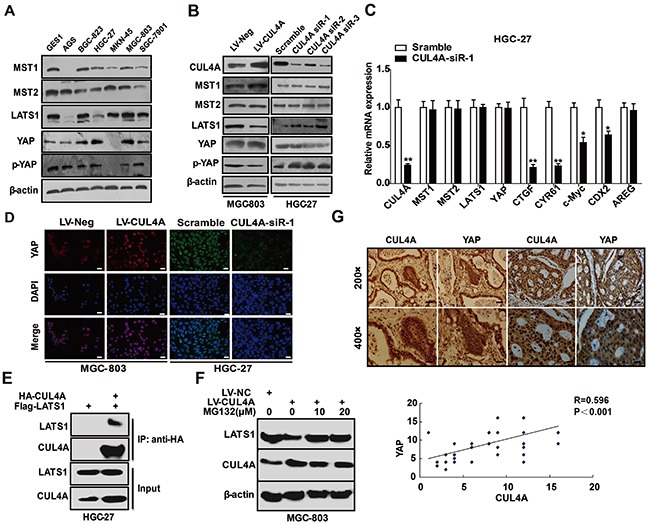
CUL4A regulates Hippo-YAP signaling **A.** Western blotting results show that the Hippo-YAP signaling proteins, MST1/2, LATS1, YAP, and p-YAP, are expressed in GC cell lines and at different levels than in the GES-1 cell line. **B.** Knockdown of CUL4A resulted in the upregulation of LATS1 and p-YAP protein levels, but had no effect on MST1/2 and total YAP protein levels in HGC-27 cells. Conversely, opposite effects were observed in MGC-803 cells when CUL4A was overexpressed. **C.** Quantitative PCR results indicated that after knocking down CUL4A, Hippo-YAP target gene mRNAs, such as for CTGF, CYR61, CDX2 and c-Myc, were decreased in HGC-27 cells. MST1/2, LATS1 and YAP mRNA levels were unchanged. **D.** Immunofluorescence results for YAP in GC cells transfected with CUL4A-siR-1 or infected with lentivirus overexpressing CUL4A. **E.** Flag-LATS1 plasmid was transfected into HGC-27 cells with or without HA-CUL4A plasmids. IPs were performed 36 h after transfection using anti-HA affinity gel beads, and IP samples were immunoblotted. **F.** CUL4A-overexpression MGC-803 cells were treated with MG132 for 6 hours and analyzed for LATS1 expression. **G.** The expression of CUL4A and YAP in 50 GC tissues was analyzed by IHC. Each bar represents the mean±SD of three independent experiments. ^*^p-value <0.05, ^**^p-value <0.01.

The Hippo signaling pathway, which regulates the YAP-TEAD complex, plays an important role in tumorigenesis [19]. The Hippo-YAP signaling pathway is involved in the development, progression and metastasis of human GC [20, 21]. Herein, we investigated whether and how CUL4A regulated Hippo-YAP signaling. We showed by western blotting that major Hippo-YAP signaling proteins, including MST1/2, LATS1, YAP, and P-YAP, are expressed in the 6 GC cell lines and in the GES-1 cell line (Figure 4A). We investigated the effects of knocking down CUL4A or overexpressing CUL4A on proteins of the Hippo-YAP signaling pathway by western blotting. The downregulation of CUL4A in HGC-27 cells using a set of 3 siRNAs resulted in an upregulation of LATS1 and P-YAP levels but did not affect MST1/2 and total YAP levels. Opposite effects were observed in MGC-803 cells when CUL4A was overexpressed (Figure 4B). Quantitative PCR results showed that after knocking down CUL4A in HGC-27 cells, the mRNAs of some Hippo-YAP target genes, such as CTGF, CYR61, CDX2, and c-Myc were reduced, but others such as MST1/2, LATS1, and YAP were unaffected (Figure 4C). Furthermore, immunofluorescence results demonstrated that the nuclear distribution of YAP was reduced after knocking down CUL4A and was enhanced after overexpressing CUL4A (Figure 4D).

LATS1 is ubiquitinated and degraded by several E3 ubiqutin ligases [22]. Thus we examined whether LATS1 is a binding partner of CUL4A and hypothesized that CUL4A overexpression might enhance the proteasomal degradation of LATS1. Our data from co-immunoprecipitation (co-IP) assays showed that CUL4A interacts with LATS1 in HGC-27 cells (Figure 4E). Moreover, overexpressing CUL4A, with MG-132 (10 and 20 μmol/L), a cell permeable proteasome inhibitor, for 6 h restored normal LATS1 protein levels, providing evidence that CUL4A promotes the proteasomal degradation of LATS1 in MGC-803 cells (Figure 4F).

These findings suggest that CUL4A might regulate YAP in GC tissues; therefore, we tested whether their expression were correlated in GC samples. Indeed, we observed that CUL4A and YAP protein levels were highly correlated in GC samples (Figure 4G, *n*=50, R=0.596, P < 0.001). These results suggest that CUL4A is involved in the inactivation of the Hippo signaling pathway.

### CUL4A is a direct target of miR-9 and miR-137

**Figure 5:**
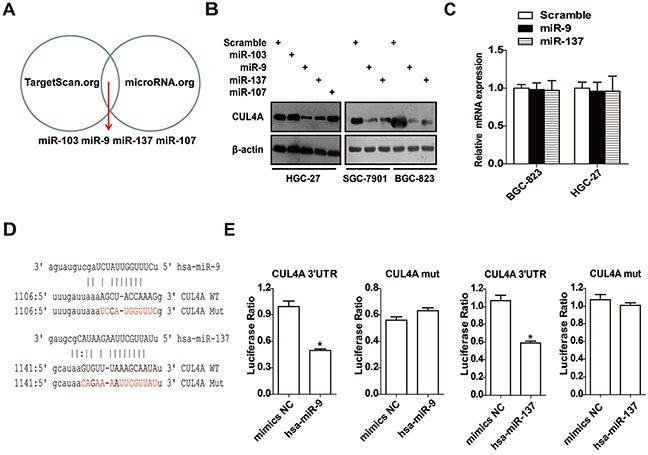
CUL4A is a direct target of miR-9 and miR-137 **A.** Four miRNAs were computationally predicted using two independent miRNA databases. **B.** Western blotting results showed that the overexpression of miR-9 and miR-137 significantly reduced CUL4A protein levels in HGC-27, SGC-7901 and BGC-823 cells; whereas, miR-103 and miR-107 had no effect on CUL4A protein levels. **C.** Quantitative PCR results of CUL4A mRNA levels after transfecting GC cells with miR-9 and miR-137 mimics. **D.** Quantitative PCR products containing predicted wild-type or mutant CUL4A 3′-UTR miRNA binding sites were inserted into luciferase reporter plasmids. **E.** In luciferase activity assays, miR-9 and miR-137 suppressed luciferase activity of the wild-type but not mutant CUL4A 3′-UTR constructs in 293 cells. Each bar represents the mean±SD of three independent experiments. ^*^p-value <0.05.

The mechanisms that regulate CUL4A expression have been largely uncharacterized. We identified putative miRNAs that might regulate CUL4A using bioinformatics methods. Four miRNAs, including miR-103, miR-107, miR-9, and miR-137 (Figure 5A), were predicted using two independent miRNA databases: TargetScan (http://www.targetscan.org/) and microRNA.org (http://www.microrna.org/). Western blotting results showed that overexpressing miR-9 and miR-137 significantly reduced CUL4A protein expression in HGC-27 cells; whereas, overexpressing miR-103 and miR-107 had no effect on CUL4A protein expression (Figure 5B). Moreover, overexpression of miR-9 and miR-137 significantly reduced CUL4A protein levels in two other cell lines, SGC-7901 and BGC-823 (Figure 5B). Quantitative RT-PCR results demonstrated that overexpressing these two miRNAs had no effect on CUL4A mRNA levels (Figure 5C).

To determine whether miR-9 and miR-137 repressed CUL4A expression by targeting its 3′-UTR, wherein the predicted binding sites were disrupted, were cloned into luciferase reporter vectors and tested using luciferase activity assays (Figure 5D). Luciferase expression from the wild-type but not from mutant 3′-UTR constructs was significantly suppressed by miR-9 and miR-137 (Figure 5E). These data support that the CUL4A 3′-UTR is a direct target of miR-9 and miR-137 in GC cells.

### miR-9 and miR-137 regulate Hippo-YAP signaling

**Figure 6:**
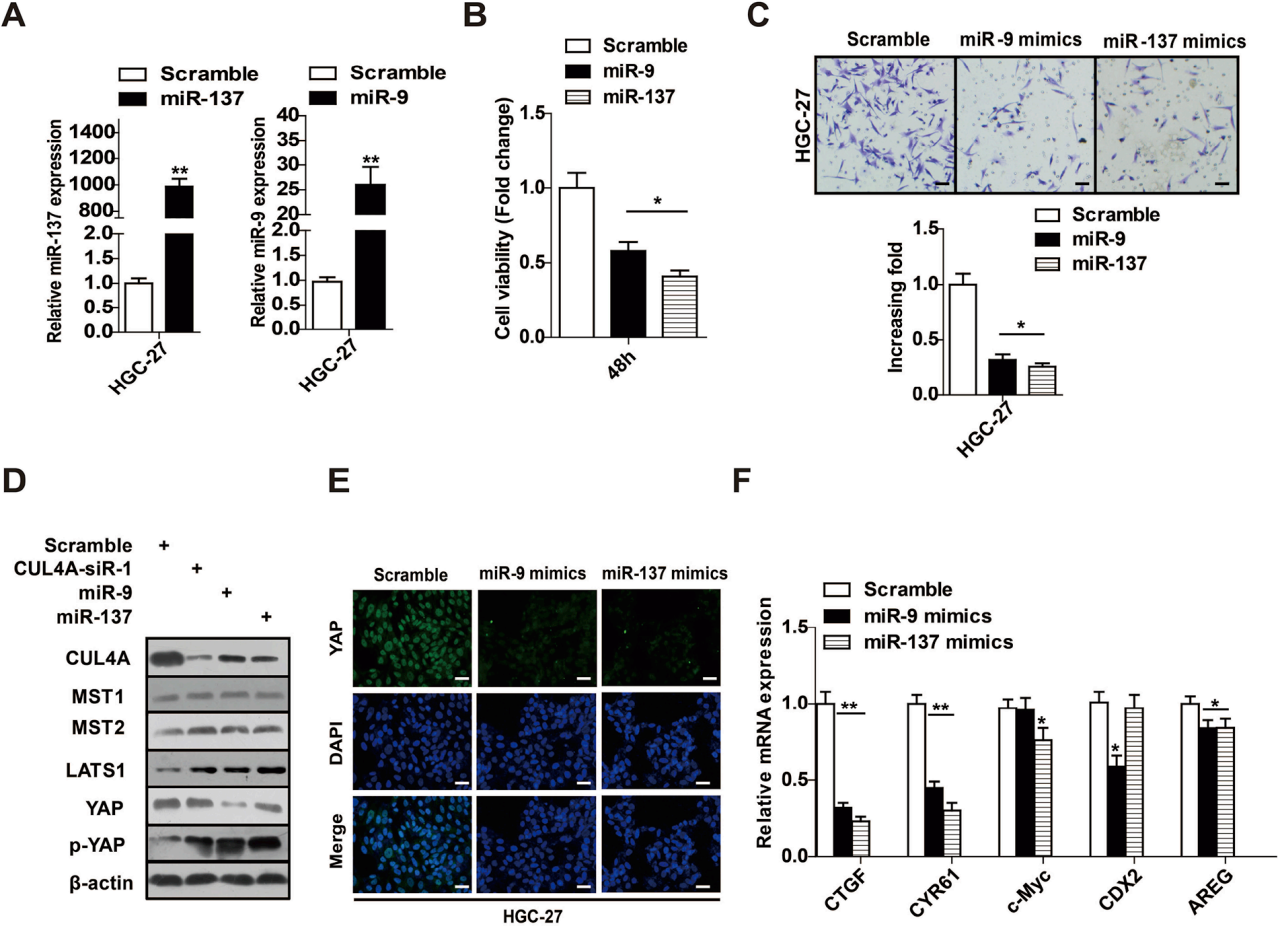
miR-9 and miR-137 regulate the CUL4A-LATS-Hippo signaling pathway **A.** Mature miR-9 and miR-137 levels were measured byqPCR after transfecting with miRNA mimics. **B.** CCK-8 assay results of HGC-27 cells after transfecting with miR-9 and miR-137 mimics. **C.** miR-9 and miR-137 inhibited HGC-27 cell invasion in Transwell chamber assays. **D.** Western blotting results ofthe levels of proteins in CUL4A-LATS1-Hippo signaling pathway in HGC-27 cells after transfecting with CUL4A-siR-1, miR-9 or miR-137, respectively. **E.** Immunofluorescence results of YAP in GC cells transfected with miR-9 and miR-137 mimics. **F.** Levels of mRNA for the indicated genes in the Hippo signaling pathway were decreased by miR-9 and miR-137 in HGC-27 cells. Each bar represents the mean±SD of three independent experiments. ^*^p-value <0.05, ^**^p-value <0.01.

**Figure 7:**
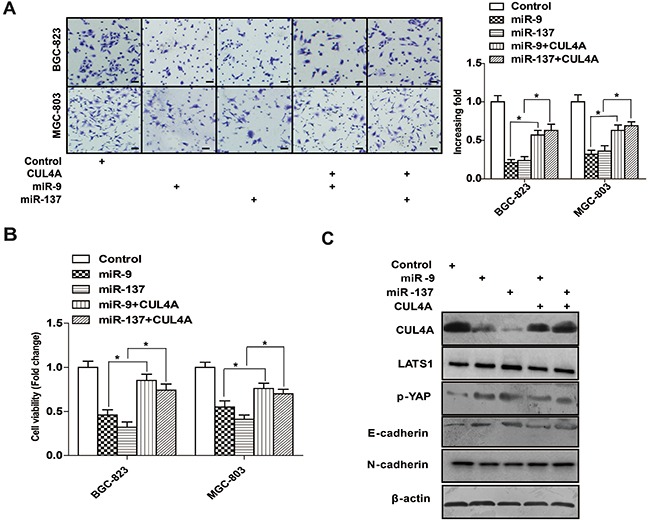
Overexpressing 3′-UTR-less CUL4A rescues miR-9/137-mediated inhibition of cell proliferation and invasion **A.** Overexpression of CUL4A missing its 3′-UTR rescued miR-9/137-mediated inhibition of MGC-803 and BGC-823 cell invasion in Transwell chamber assays. **B.** Overexpression of CUL4A missing its 3′-UTR rescued miR-9/137-mediated inhibition of MGC-803 and BGC-823 cell proliferation in CCK-8 assays. **C.** Western blotting results of the levels of the indicated proteins in HGC-27 cells co-transfected with 3′-UTR-less CUL4A and miR-9 or miR-137. Each bar represents the mean±SD of three independent experiments. ^*^p-value <0.05.

Several studies have demonstrated that miR-9 and miR-137 are decreased in human GC tissues and cell lines, suggesting that they function as tumor suppressors [23, 24]. This is supported by our observation that overexpressing miR-9 and miR-137 suppressed the proliferation and invasion of GC cells *in vitro* (Figure 6A, 6B, 6C). Recently, several studies have demonstrated that miRNAs can regulate Hippo signaling [25, 26]. Our findings suggest that miR-9 and miR-137 might regulate Hippo-YAP signaling in GC cells by targeting CUL4A. As shown by western blotting in Figure 6D, overexpressing miR-9 and miR-137 in HGC-27 cells resulted in the upregulation of LATS1 and p-YAP and downregulation of CUL4A, but no change in MST1/2. Interestingly, both miRNAs reduced the total protein level of YAP in HGC-27 cells (Figure 6D). In addition, immunofluorescence results showed that the nuclear distribution of YAP was reduced after transfecting HGC-27 cells with miR-9 and miR-137 mimics (Figure 6E). Moreover, qPCR results demonstrated that miR-9 decreased the expression of downstream targets of the Hippo pathway, such as CTGF, CYR61, CDX2 and AREG, but not of c-Myc (Figure 6F). In addition, miR-137 decreased the expression of CTGF, CYR61, AREG, and c-Myc, but not of CDX2 (Figure 6F). Importantly, co-transfecting miR-9 or miR-137 with a CUL4A gene construct missing its 3′-UTR into MGC-803 or BGC-823 cells rescued miR-9/137-mediated inhibition of GC cell invasion (Figure 7A), cell proliferation (Figure 7B) and EMT (Figure 7C) through the Hippo pathway (Figure 7C).

**Figure 8:**
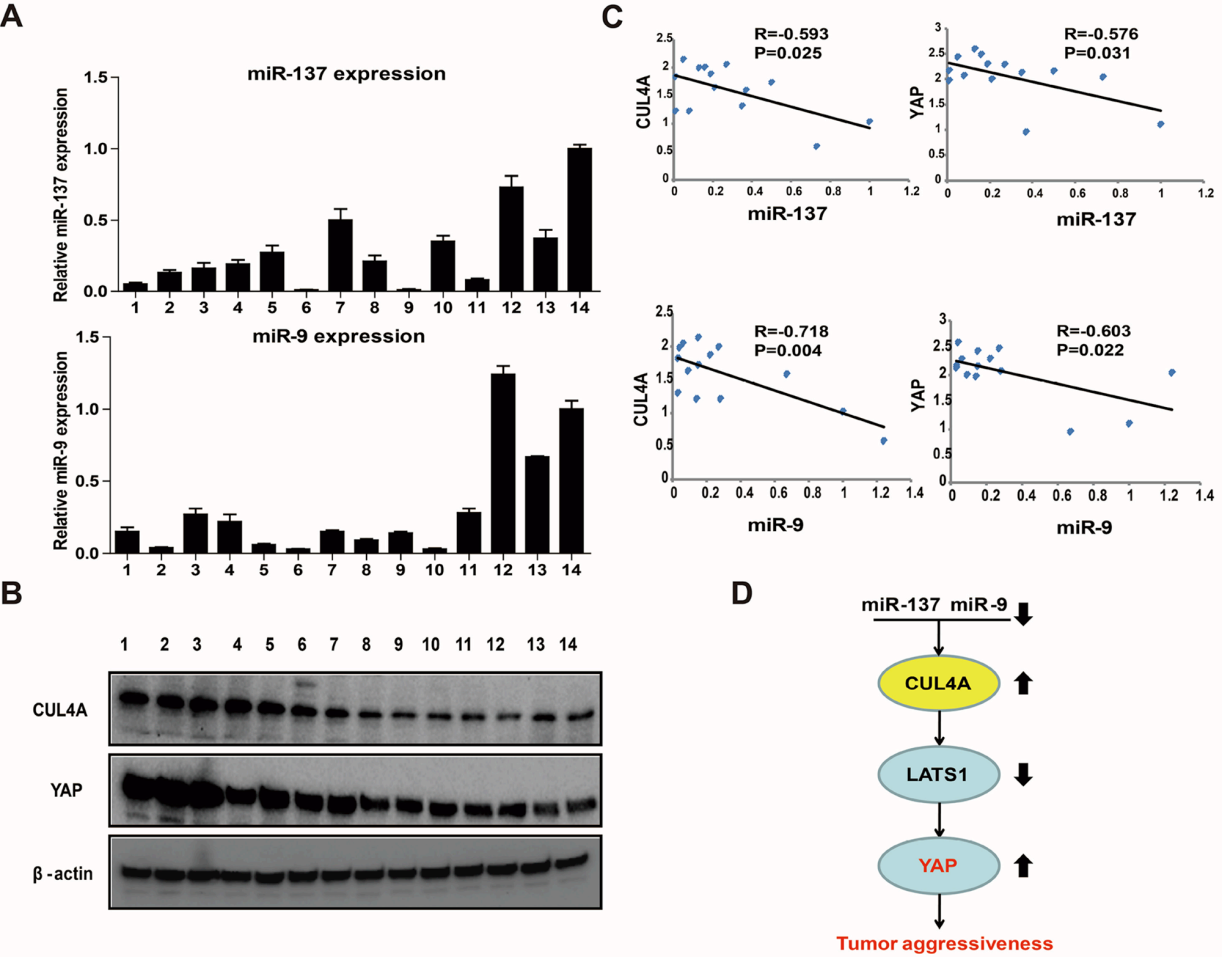
Clinical relevance of the miR-9/137-CUL4A-Hippo signaling axis in fresh GC tissues **A.** The relative levels of miR-9 and miR-137 expression in 14 fresh GC tissues were determined by qPCR. **B.** Western blotting results of CUL4A and YAP protein levels in 14 fresh GC tissues. **C.** Statistical analysis suggested an inverse correlation between miR-137 and miR-9 expression and CUL4A and YAP protein expression, respectively. **D.** Schematic of the miR-137/9-CUL4A-Hippo signaling axis in GC. Each bar represents the mean±SD of three independent experiments. (Arrows represent up or down regulation in gastric cancer cells)

We detected the expression of miR-9 and miR-137 in addition to CUL4A and YAP proteins in 14 fresh GC tissues, which included seven clinical stage I-II and seven clinical stage III-IV samples (Figure 8A, 8B). We observed that the expression of miR-137 was inversely correlated with protein levels of CUL4A (Figure 8C, R=−0.593, P=0.025) and YAP (Figure 8C, R=−0.576, P=0.031). An inverse correlation was also observed between miR-9 expression and CUL4A (Figure 8C, R=−0.718, P=0.004) and YAP (Figure 8C, R=−0.603, P=0.022) protein levels. Taken together, these results suggest that a miR-9/137-CUL4A-Hippo signaling axis plays a vital role in the development and progression of GC (Figure 8D).

## DISCUSSION

In the present study, we have demonstrated the importance of CUL4A to gastric tumorigenesis. Knocking down CUL4A inhibited GC cell proliferation and EMT *in vitro* and *in vivo*. Conversely, overexpression of CUL4A had the opposite effect *in vitro*. We also showed that CUL4A mediated its effects in GC cells by regulating the LATS1-Hippo signaling pathway. Moreover, we showed that miR-9 and miR-137 targeted CUL4A in GC cells, thereby indirectly regulating the LATS1-Hippo signaling pathway and promoting cell proliferation and invasion. Importantly, a significant correlation among miR-137/9, CUL4A and YAP was observed in GC samples. Hence, a miR-137/9-CUL4A-Hippo signaling axis that results in the inactivation of Hippo promotes GC proliferation and invasion.

A growing body of evidence indicates that CUL4A can function as an oncogene in different tumor types, and thus can be used as a prognostic molecular biomarker for different cancers [7, 18]. Yang et al. demonstrated that overexpression of CUL4A induced lung tumorigenesis in a conditional CUL4A transgenic mouse model [27]. CUL4A also contributes to basal-like breast cancers via activation of the Ras signaling pathway [28]. However, the functions of CUL4A and the mechanisms that regulate its expression in GC have been less well characterized. In our study, we found that the expression of CUL4A significantly correlated with GC patients' TNM classification and lymph node metastasis. Furthermore, patients with higher levels of CUL4A had lower survival rates than patients with lower levels of CUL4A, suggesting that CUL4A may represent a novel predictor for GC prognosis and survival. However, CUL4A expression was not statistically different based on tumor differentiation, indicating that CUL4A may not participate in the differentiation process. Knocking down CUL4A significantly reduced GC cell proliferation, EMT, and invasion *in vitro* and *in vivo*; conversely, CUL4A overexpression led to opposite results *in vitro*. CUL4A ubiquitinates and degrades p21 and p27 in various cell lines [5, 29]. Here, we also demonstrated that CUL4A regulated p21 and p27 expression in GC cell lines. Therefore, our results suggest that in GC, CUL4A functions as an oncogene.

As a tumor suppressor pathway, therapeutic targeting of the Hippo signaling pathway has been considered recently [19, 30]. The Hippo signaling pathway includes a core suppressor kinase, such as MST1/2 and LATS1/2, whose expression can be reduced by ubiquitination and methylation [22, 31–35]. Upon Hippo inactivation, the transcriptional co-activator YAP translocates into the nucleus and combines with the TEAD family of proteins to activate downstream gene expression, including for CTGF, CYR61, CDX2, AREG, c-Myc, and survivin [36–38]. Hippo inactivation induces cell proliferation, apoptosis, EMT and drug resistance [39–41]. In GC, 3,3-diindolylmethane (DIM) has antitumor activity by regulating the Hippo-YAP signaling pathway [42]. Furthermore, VGLL4 directly competes with YAP for binding to TEADs, thus suppressing GC cell growth *in vitro* and *in vivo* [36]. However, the mechanism of Hippo inactivation in GC remains unclear. In the present study, we showed that reduced CUL4A expression in GC cell lines activated the LATS1-Hippo signaling pathway without activating MST1/2. Conversely, increased CUL4A expression led to opposite effects. Furthermore, CUL4A interacted with LATS1 and enhanced LATS1 protein proteasomal degradation, suggesting that CUL4A is a critical mediator of Hippo-YAP signaling inactivation in GC. A recent study demonstrated that DCAF1, an E3 ubiquitin ligase of the CRL4^DCAF1^ complex inhibited kinases of the Hippo signaling pathway by directly binding to and ubiquitinating LATS1/2 in NF2-mutant tumors [43]. Interestingly, CUL4A is a core component of the CRL4 complex, whereas its N-terminus associates with a cullin-specific adaptor protein to recruit a large family of substrate proteins [44, 45]. These observations may explain how CUL4A inhibits LATS1 kinases; however, the exact mechanisms warrant further investigation.

Although several studies have investigated the function of CUL4A in different cancer types, less is known about the mechanisms that regulate CUL4A expression. Many studies have shown that miRNAs can regulate various physiological processes [46–48]. Recently, some members of the Cullin family of proteins were shown to be regulated by miRNAs. For instance, downregulation of miR-141 increases CUL3 expression in Hirschsprung's disease [49], and miR-19 targets CUL5 to regulate proliferation and invasion of cervical cancer cells [50]. Here, using bioinformatics and luciferase reporter assays, we identified that the miRNAs, miR-9 and miR-137, directly target the 3′-UTR of CUL4A transcripts. Furthermore, we verified that miR-9/137 indirectly regulated LATS1-Hippo signaling and suppressed GC cell proliferation and invasion by directly targeting CUL4A. The miR-9/137-CUL4A-Hippo signaling axis we have described here may have important clinical implications, since we also observed highly significant correlations among miR-9/137, CUL4A and YAP in GC tissue samples.

In summary, our study has demonstrated that CUL4A upregulation is associated with poor prognosis in patients with GC. We also demonstrated that perturbations to a miR-9/137-CUL4A-Hippo signaling axis contributed to gastric tumorigenesis. Our characterization of this signaling pathway contributes to a better understanding of the development and progression of GC, and may provide novel therapeutic targets for the future treatment of GC.

## MATERIALS AND METHODS

### Tissue samples and cell culture

Paraffin-embedded GC tissue samples from 124 GC patients were collected and archived at the First Affiliated Hospital of Nanchang University between January 2008 and December 2010. The clinicopathological findings for each sample are summarized in Supplementary Table S1. Twenty noncancerous gastric tissues (controls) were also collected and archived. None of the patients included in our study received preoperative chemotherapy. A total of 18 fresh GC tissues and paired noncancerous gastric mucosal tissues were immediately snap-frozen in liquid nitrogen and stored at −80°C until their use in qPCR and western blotting assays (Supplementary Table S2). All patients provided written informed consent to participate in the study. GC (SGC-7901, AGS, BGC-823, MGC-803, MKN-45, HGC-27), GES-1 and HEK293 cell lines were cultured in RPMI 1640 (HyClone, Logan, UT, USA) supplemented with 10% fetal bovine serum (FBS; Sigma, St. Louis, MO, USA) and grown in a humidified 37°C incubator with 5% CO_2_.

### Cell transfection and infection

Three short hairpin RNAs (CUL4A siR-1/2/3) were designed: CUL4A siR-1, 5′-CUGCUAUCGUCAGA AUAAUTT-3′; CUL4A siR-2, 5′-CCAUCUGGGAUA UGGGAUUTT-3′; CUL4A siR-3, 5′-GCAAAGCAU GUGGAUUCAATT-3′.

HA-CUL4A plasmid, Flag-LATS1 plasmid, miRNA mimics and their respective negative controls were purchased from Genepharma (Shanghai, China). Cells were grown to 50-70% confluence and transfected using Lipofectamine 2000 (Invitrogen, Waltham, MA, USA) following the manufacturer's protocol. Short hairpin RNAs targeting CUL4A and the full-length CUL4A gene were subcloned into the lentiviral expression vector, GV248 (Genepharma). An empty GV248 vector was used as the negative control. After 48 h of transfection or 96 h of infection, the efficiency of knockdown or overexpression was assayed by real-time PCR and western blotting.

### Immunohistochemistry and immunofluorescence

IHC and immunofluorescence (IF) were performed following previously described methods [11]. IHC analysis was performed on 124 clinical GC tissue sections. The immunostaining index was independently reviewed and scored by two pathologists based on the intensity of staining and the proportion of positively stained tumor cells. The intensity of staining was graded according to the following scale: 1 (no staining), 2 (weak staining; light yellow), 3 (moderate staining; yellow–brown), 4 (strong staining; brown). Positively stained tumor cells were graded according to the following scale: 0 (no positively stained cells), 1 (<10%), 2 (10%–35%), 3 (35%-75%), or 4 (>75% of positively stained cells). The immunostaining index (SI) was calculated as the proportion of positively stained tumor cells × the staining intensity score. Tumors with SI values between 0 and 6 were considered negative and between 8 and 16 were considered positive.

### Quantitative real-time RT-PCR

Total RNA was extracted from GC tissues and cell lines using TRIzol reagent (Invitrogen) and reverse transcription was performed using a reverse transcription kit (Invitrogen), following the manufacturers' instructions. Quantitative real-time PCR (qRT-PCR) reactions were performed on an ABI 7500 real-time fast PCR system (Applied Biosystems, Waltham, MA, USA). The primers used in this study are listed in Supplementary Table S3. The levels of miRNAs and mRNAs were normalized by U6 and GAPDH levels, respectively.

### Immunoblotting and IP

Total protein was extracted from GC tissues and cells by lysing in ice-cold lysis buffer. The proteins were electrophoresed on an SDS-PAGE gel, transferred to a PVDF membrane (Millipore, Danvers, MA, USA), and probed with a primary antibody targeted against CUL4A (Abcam, Cambridge, MA, USA), p21 (Abcam), p27 (Abcam), E-cadherin (Abcam), N-cadherin (Abcam), MST1 (Cell Signaling, Danvers, MA, USA), MST2 (Cell Signaling), LATS1 (Cell Signaling), YAP (Cell Signaling), p-YAP (Cell Signaling), vimentin (Cell Signaling), fibronectin (Cell Signaling) or β-actin (Cell Signaling). After incubating with the primary antibody, membranes were washed with TBS/0.05% Tween-20 (TBST) and incubated with a horseradish peroxidase-conjugated secondary antibody (Cell Signaling) for 1 h at room temperature. After washing 3 times with (TBST) for 15 min, the membranes were developed using an ECL plus western blotting detection system.

For IP assays, HGC-27 cells were transfected with the Flag-LATS1 plasmid or co-transfected with Flag-LATS1 and HA-CUL4A plasmids, then treated with MG132 (10 μmoL) for 6 h, and lysed with the lysis buffer. Cell lysates were incubated with 5 μL of anti-HA beads (Catalog Number E6779, Sigma, USA) at 4°C for 4 h, then centrifuged and washed with RIPA buffer for 3 times. IP samples were immunoblotted in subsequent experiments.

### *in vitro* cell proliferation assays

MTT, CCK-8, and colony formation assays were used to determine the biological effect of CUL4A and miR-9/137 on GC cell proliferation, following previously described methods [15, 51].

### *in vitro* cell migration and invasion assays

For invasion assays, 5.0×10^4^ cells were seeded in the upper well of a Matrigel coated chamber with serum-free media (BD Biosciences, Franklin Lakes, NJ, USA). Complete growth medium was added to the lower well of each chamber. After 48 h of incubation at 37°C, cells that had migrated or invaded to the lower well of the chamber were fixed and stained in dye solution containing 20% methanol violet and 0.1% crystal, and imaged using a BH-2 inverted microscope (Olympus, Tokyo, Japan). Cell counts are expressed as the mean number of cells per field of view and were normalized to the negative control group. Three independent experiments were performed and the data are presented as mean ± standard deviation (SD).

### Xenograft studies in athymic nude mice

Four to five weeks old BALB/c nude mice were purchased from the Center of Experimental Animal of Guangzhou University of Chinese Medicine (Guangzhou, China). BALB/c nude mice were randomly divided into 3 groups (*n*=5/group). HGC-27 cells (1.0×10^7^) were infected with an empty lentivirus (control) or a lentivirus encoding CUL4A shRNAs. Tumor volumes were measured every 3 days with a caliper and calculated using the following formula: (L×W)^2^/2 (L indicates the length-diameter and W the width-diameter of the tumor).

### Luciferase reporter plasmids and assays

Predicted miRNA binding regions for miR-9 and miR-137 in the 3′-UTR of CUL4A were subcloned into the pMIR-REPORT Luciferase miRNA expression vector (pLuc, Ambion, Waltham, MA, USA). Mutants of the binding sites were used as the negative control. The following primers were used: CUL4A-3UTR-HF: 5′-AATTCTAGGCGATCGCTCGAGGCAGGGACGAT CCTTGTTCT-3′; CUL4A-3UTR-HR: 5′-GCGGCCGC TCTAGGTTTAAACAGACCACATATCATGGAACT CAT-3′; CUL4A-miR9-MR: 5′-AAATTCGAAACCATGG ATTTAATCAAAATGAAACATGC-3′; CUL4A-miR9-MF: 5′-TAAATCCATGGTTTCGAATTTTGATCATGG CATAAG-3′; CUL4A-miR137-MR:5′-AGAAAAATAAC GAATTTTCTGTTATGCCATGATC-3′; CUL4A-miR137-MF: 5′-GCATAACAGAAAATTCGTTATTTTTCTGGAATA TACC-3′. HEK293 cells were seeded in 24-well plates at a density of 1×10^5^ cells per well and transiently transfected with wild type or mutant luciferase reporter plasmids (miR-9, miR-137 and the negative control) at a final concentration of 50 nM. Following 48 h of incubation, luciferase activity was measured using a dual-luciferase reporter system (Promega, Fitchburg, WI, USA).

### Statistical analysis

Data were analyzed using the statistical software, SPSS 18.0 (IBM, Armonk, NY, USA). Data were expressed as the mean±SD of at least three independent experiments. The difference between groups was determined by an unpaired two-tailed Student's t-test. A p-value < 0.05 was considered statistically significant.

## SUPPLEMENTARY FIGURES AND TABLES


