# Supplementary material for: Cullin 4A (CUL4A), a direct target of miR-9 and miR-137, promotes gastric cancer proliferation and invasion by regulating the Hippo signaling pathway
Source: Oncotarget. 2016 Jan 28;7(9):10037–50. doi: 10.18632/oncotarget.7048 (PMC4891102; doi:10.18632/oncotarget.7048)
Supplement: Supplementary file 1 [file oncotarget-07-10037-s001.pdf]

SUPPLEMENTARY TABLES

Supplementary Table S1: The relationship between CUL4A expression and clinicopathological parameters in 124 GC patients (*P<0.05)

Groups	CUL4A protein expression (*n* = 124)			p Value
	positive	negative	No.	
Age (years)				
<60	30	22	52	0.715
≥60	38	34	72	
Gender				
Male	36	29	65	1.000
Female	32	27	59	
Lauran				
Intestinal	45	32	77	0.354
Nonintestinal	23	24	47	
Differentiation				
Low	28	20	48	0.582
Moderate or well	40	36	76	
Tumor size<cm>				
≤ 4	20	21	41	0.443
>4	48	35	83	
pT stage				
pT1+pT2	16	24	40	0.033*
pT3+pT4	52	32	84	
Lymph node metastasis				
N0+N1	17	29	46	0.003*
N2+N3	51	27	78	
UICC stage				
I/II	18	26	44	0.025*
III/IV	50	30	80	
Total	68	56	124	

Supplementary Table S2: Clinicopathological characteristics of the 18 gastric cancer patients

Patient no.	Age(years)	Sex	TNM classification	Clinical stage	Tumour size(cm^3)	Histological type (differentiated)
**1**	53	F	$T_2N_0M_0$	I	2 × 1 × 1	Moderate
**2**	42	F	$T_3N_2M_0$	III	6 × 4 × 3	Well
**3**	68	M	$T_3N_0M_0$	II	3 × 3 × 1	Poor
**4**	44	M	$T_4N_2M_0$	III	6 × 5 × 8	Moderate
**5**	51	F	$T_3N_2M_0$	III	4 × 6 × 5	Well
**6**	47	M	$T_3N_0M_0$	II	1 × 3 × 2	Well
**7**	56	F	$T_4N_3M_0$	III	8× 4 × 3	Poor
**8**	60	M	$T_2N_0M_0$	I	2 × 1 × 1	Moderate
**9**	56	F	$T_3N_0M_0$	II	2× 3 × 0.5	Well
**10**	49	M	$T_2N_3M_0$	III	5× 5 × 3	Poor
**11**	60	F	$T_3N_2M_0$	III	4.5 × 2 × 3	Moderate
**12**	55	M	$T_3N_3M_0$	III	8× 4 × 6	Poor
**13**	49	F	$T_1N_2M_0$	II	1× 2 × 1.5	Moderate
**14**	57	F	$T_3N_0M_0$	II	4 × 1 × 4.5	Moderate
**15**	62	M	$T_3N_2M_0$	III	6× 5 × 2	Poor
**16**	58	F	$T_3N_0M_0$	II	3.5 × 3 × 1.5	Well
**17**	49	F	$T_4N_2M_0$	III	2 × 3 × 4	Moderate
**18**	44	M	$T_2N_1M_0$	II	2 × 3 × 2	Poor

M, male; F, female.

Supplementary Table S3: Primers

MST1	F: GTGATGAGATGGGCACTGTC	R: TCCTCTGCATTGATCACCAT
MST2	F: CTGGTCCCTTGGCATTACTT	R: GGACCAAAGTTCTGGCTTTC
LATS1	F: GGCTTCAGATGGACACACGA	R: GCTCTCCGCTCTAATGGCTT
YAP	F: GCATGATCTGCCCTAAGGC	R: TGACCGCCGAGTACACCAT
c-Myc	F: CCCTCCACTCGGAAGGACTA	R: GCGGTGCATTTTCGGTTGT
CTGF	F: CAAGGGCCTCTTCTGTGACTT	R: GGTACACCGTACCACCGAAG
CYR61	F: CGCCTTGTGAAAGAAACCCG	R: GGTTCGGGGGATTTCTTGGT
CDX2	F: GAGTGGTGTACACGGACCAC	R: CTCCTTTGCTCTGCGGTTCT
AREG	F: TGTCGCTCTTGATACTCGGC	R: ATGGTTCACGCTTCCCAGAG
CUL4A	F: GCAACAAAGAAGCCACAGACG	R: CGACTGAGGCACTTTTCCCA
miR-9	F: GGGTCTTTGGTTATCTAGC	R: TGCGTGTCGTGGAGTC
miR-137	F: GCGUUAUUGCUUAAGAAUAC	R: CAGTGCAGGGTCCGAGGT
U6	F: AGAGCCTGTGGTGTCCG	R: CATCTTCAAAGCACTTCCCT
GAPDH	F: GGCATCCTGGGCTACACTGA	R: GAGTGGGTGTCGCTGTTGAA
